# Comparing a virtual reality head-mounted display to on-screen three-dimensional visualization and two-dimensional computed tomography data for training in decision making in hepatic surgery: a randomized controlled study

**DOI:** 10.1007/s00464-023-10615-8

**Published:** 2024-03-08

**Authors:** Anas Amin Preukschas, Philipp Anthony Wise, Lisa Bettscheider, Micha Pfeiffer, Martin Wagner, Matthias Huber, Mohammad Golriz, Lars Fischer, Arianeb Mehrabi, Fabian Rössler, Stefanie Speidel, Thilo Hackert, Beat Peter Müller-Stich, Felix Nickel, Hannes Götz Kenngott

**Affiliations:** 1 Department of General, Visceral and Transplantation Surgery, University of Heidelberg, Im Neuenheimer Feld 672, 69120 Heidelberg, Germany; 2 Department of General, Visceral and Thoracic Surgery, University Medical Center Hamburg-Eppendorf, Martinistraße 52, 20246 Hamburg, Germany; 3 Institute for Anthropomatics and Robotics, Karlsruhe Institute of Technology, Kaiserstrasse 12, 76131 Karlsruhe, Germany; 4 Department for Translational Surgical Oncology, National Center for Tumor Diseases, Fiedlerstraße 23, 01307 Dresden, Germany; 5 Department of Surgery, Hospital Mittelbaden, Balgerstrasse 50, 76532 Baden-Baden, Germany; 6 Division of Abdominal Surgery, Clarunis Academic Centre of Gastrointestinal Diseases, St. Clara and University Hospital of Basel, Petersgraben 4, 4051 Basel, Switzerland; 7 Department of Surgery and Transplantation, University Hospital of Zürich, Rämistrasse 100, 8091 Zurich, Switzerland

**Keywords:** Virtual reality, Head mounted display, Hepatic surgery training, Three dimensional visualization

## Abstract

**Objective:**

Evaluation of the benefits of a virtual reality (VR) environment with a head-mounted display (HMD) for decision-making in liver surgery.

**Background:**

Training in liver surgery involves appraising radiologic images and considering the patient’s clinical information. Accurate assessment of 2D-tomography images is complex and requires considerable experience, and often the images are divorced from the clinical information. We present a comprehensive and interactive tool for visualizing operation planning data in a VR environment using a head-mounted-display and compare it to 3D visualization and 2D-tomography.

**Methods:**

Ninety medical students were randomized into three groups (1:1:1 ratio). All participants analyzed three liver surgery patient cases with increasing difficulty. The cases were analyzed using 2D-tomography data (group “2D”), a 3D visualization on a 2D display (group “3D”) or within a VR environment (group “VR”). The VR environment was displayed using the “Oculus Rift ™” HMD technology. Participants answered 11 questions on anatomy, tumor involvement and surgical decision-making and 18 evaluative questions (Likert scale).

**Results:**

Sum of correct answers were significantly higher in the 3D (7.1 ± 1.4, *p* < 0.001) and VR (7.1 ± 1.4, *p* < 0.001) groups than the 2D group (5.4 ± 1.4) while there was no difference between 3D and VR (*p* = 0.987). Times to answer in the 3D (6:44 ± 02:22 min, *p* < 0.001) and VR (6:24 ± 02:43 min, *p* < 0.001) groups were significantly faster than the 2D group (09:13 ± 03:10 min) while there was no difference between 3D and VR (*p* = 0.419). The VR environment was evaluated as most useful for identification of anatomic anomalies, risk and target structures and for the transfer of anatomical and pathological information to the intraoperative situation in the questionnaire.

**Conclusions:**

A VR environment with 3D visualization using a HMD is useful as a surgical training tool to accurately and quickly determine liver anatomy and tumor involvement in surgery.

**Supplementary Information:**

The online version contains supplementary material available at 10.1007/s00464-023-10615-8.

Hepatic resections are often the only curative treatment options for malignant hepatic lesions [1]. They can be complex surgical procedures with considerable morbidity and mortality rates [2–6]. Thorough planning is required in a multidisciplinary team weighing radiological findings, surgical options and medical possibilities for deciding on the extent of individual resection [4, 6–9]. Training in liver surgery takes many years until an adequate level of competence is achieved [10, 11]. Decision-making in liver surgery requires detailed knowledge of the liver and vessel anatomy and its variations, as well as tumor biology and comorbidities. To determine the most beneficial approach for each patient, heterogeneous data and information from a wide range of medical disciplines must be considered [5, 6, 8, 9, 12–15]. Due to this complexity, it can be difficult for surgical novices to comprehend decision-making in hepatic surgery, which is further complicated when considering that the traditional way of determining the surgical strategy using tomography imaging data on a 2D monitor does not provide the optimal framework for decision-making in high-risk procedures and complex cases. 3D operation planning has been proven to facilitate surgical decision-making in liver surgery as it aids in identifying the unique anatomy and tumor involvement and can also assist in choosing the most adequate procedure [16–19]. In addition, many studies have shown that 3D display is superior to 2D display of tomography images in learning surgical liver anatomy [20–24] and can help correctly locate hepatic tumors and decide on the optimal extent of the hepatic resection [25]. The feasibility and benefit of 3D surgical planning has already been described and a number of commercial solutions are available [16, 26, 27].

Virtual reality (VR) head-mounted displays (HMD) revolutionize the way we interact with data, allowing for an immersive and intuitive way of gathering experience in surgery [19, 28]. As previously stated, decision of adequate therapy, in this case on the example of hepatic surgery, requires a synthesis of multiple heterogeneous datapoints from different medical disciplines, often presented in different forms of media, such as pictures, video, data tables, and free text among other modalities, often distributed across multiple data platforms [5, 6, 8, 9, 12–15]. This heterogeneity demands more intuitive ways of data presentation for the surgeon to make timely and correct decisions [6, 12, 29, 30]. VR may provide a new framework to combine surgery data and relevant clinical information. Surgical education for medical students and residents could also be improved by VR and 3D imaging technologies and training tools [31–37]. VR with HMD provides an immersive and interactive solution for individual and grouped interaction and integrated presentation of imaging and necessary clinical information [19, 29, 30, 36–41]. The necessity for tools that allow for remote interaction with patient data and with medical specialists has been increasing with the rise of specialized “expertise centres”, and the COVID-19 pandemic has only increased this demand [41–44].

The aim of this study is to explore the benefits and problems of a VR environment using a HMD as an immersive and interactive tool for training surgical novices for liver surgery and to compare it to on-screen 3D visualization and 2D-tomography data.

## Material and methods

### Patient cases

Four representative patient cases from the Department of General, Visceral, and Transplantation Surgery at Heidelberg University Hospital who underwent liver surgery were chosen for this study, of which one case was used as a training dataset in order to familiarize patients with their respective visualization method. Table 1 shows the patient vignette information and important radiological findings for the three patient cases used as test datasets. Additionally, participants had access to the patient’s most recent lab results. All patient data was anonymized before it was included in this study.

**Table 1:** Patient information vignettes of the hepatic resection cases

	Patient 1	Patient 2	Patient 3
Pathology	Intrahepatic cholangiocarcinoma	Intrahepatic cholangiocarcinoma	Hepatic metastasis of a neuroendocrine tumor
TNM	pT1 N0 M0	pT2a Nx M0	Not applicable
Stage	I	II	IV
Tumor involvement	Segment 4(a)	Segments 8, 4a+b	Segments 8+(5)
Portal vein involvement	Left portal vein	Right portal vein	Right anterior pedicle
Hepatic vein involvement	Middle and left hepatic vein	Middle hepatic vein	Middle hepatic vein
Hepatic artery involvement	None	Right and middle hepatic artery	Middle hepatic artery
Bile duct involvement	Right hepatic duct	Right and left hepatic duct	None
Arterial anomalies	Accessory left hepatic artery from the gastric artery	None	None
Hepatic vein anomalies	Accessory right inferior hepatic vein	Accessory right inferior hepatic vein	None
Theoretically feasible resections	Left hemihepatectomy, extended left hemihepatectomy	Extended right hemihepatectomy	Mesohepatectomy, extended right hemihepatectomy
Planned operation	Left hemihepatectomy	Extended right hemihepatectomy	Mesohepatectomy

### Imaging data and segmentation

**Fig. 1:**
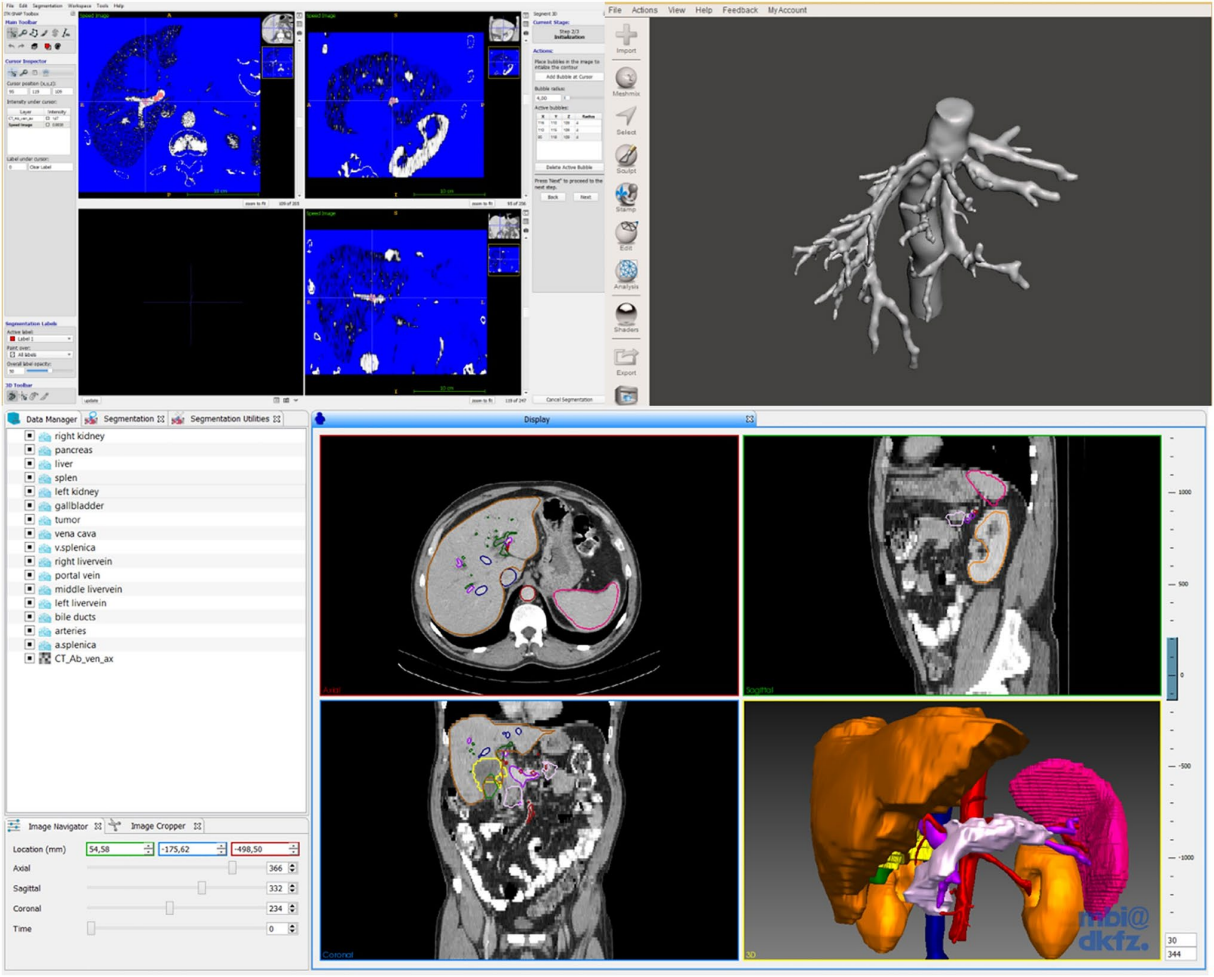
Segmentation software examples: Medical Imaging Interaction Toolkit (below), ITK-Snap (top left), MeshMixer (top right)

**Fig. 2:**
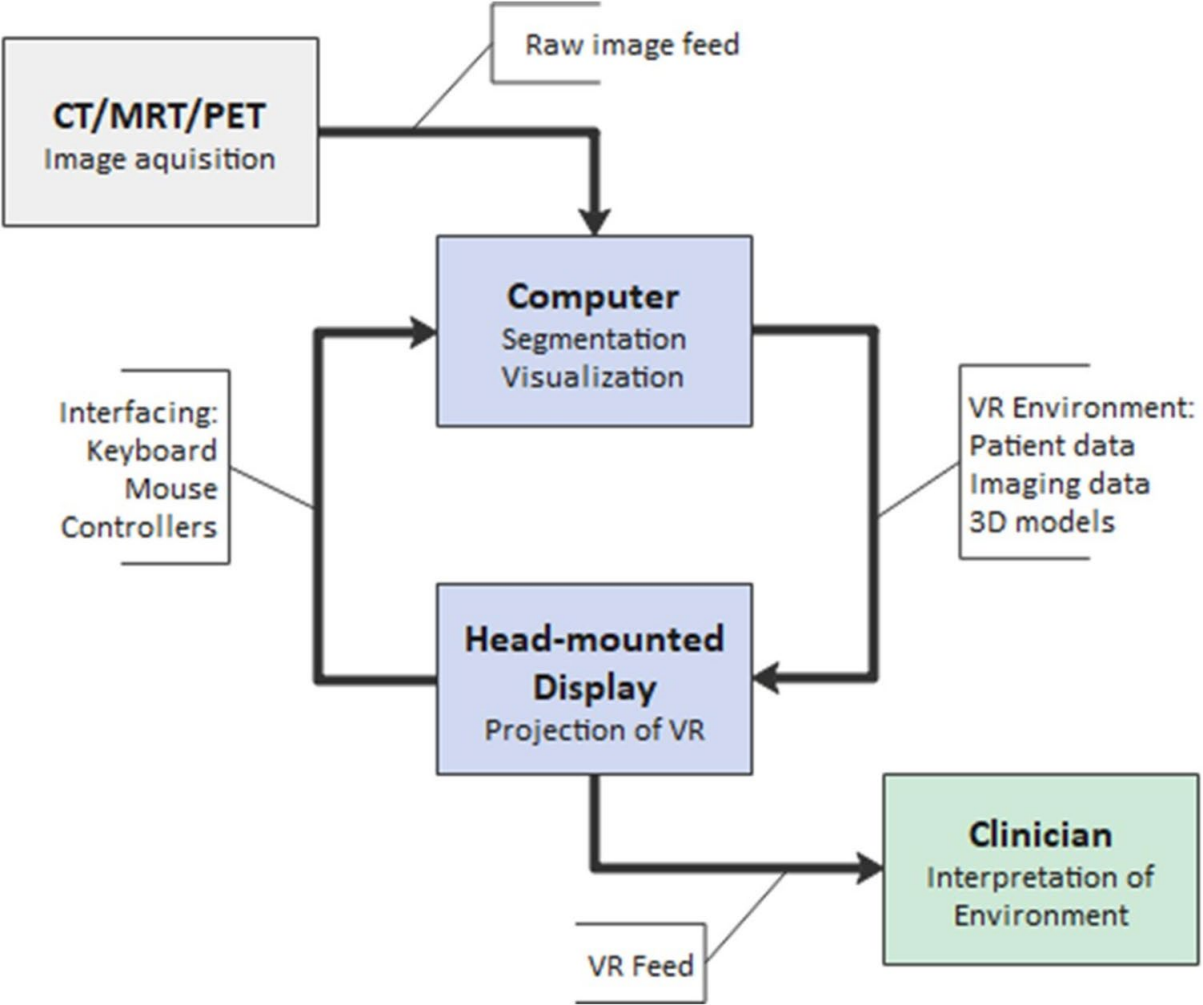
Virtual reality workflow

**Fig. 3:**
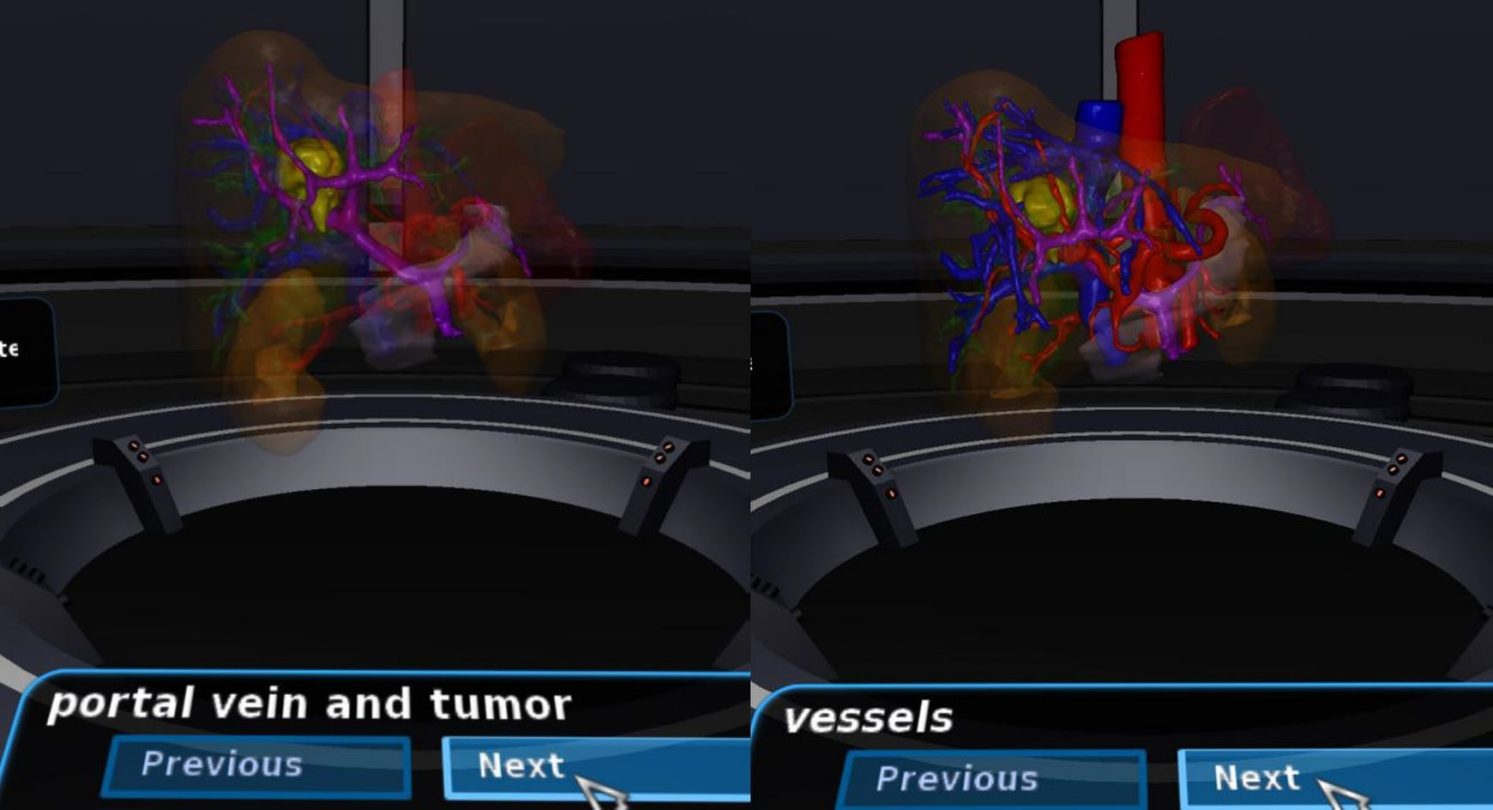
Example of Virtual Reality environment from inside the Oculus Rift®

Radiological images were anonymized and then retrieved from the Pictures Archiving and Communication System (PACS) of the Department of General, Visceral, and Transplantation Surgery at Heidelberg University Hospital in an anonymized fashion. The images complied with the Digital Imaging and Communications in Medicine (DICOM). Several open-source software applications were used for the segmentation and post-processing of the original DICOM-images. Organ surfaces were segmented semi-automatically using the Medical Imaging and Interaction Toolkit (MITK, German Cancer Research Center, Heidelberg, Germany, www.mitk.org). Vessels and bile ducts were segmented semi-automatically using ITK-snap (www.itksnap.org). Segmentations were performed using images in the portal venous phase. The segmentation of the arteries was performed using images in the arterial phase. To align the arterial with the portal venous images, they were registered using 3D Slicer (www.slicer.org). The post-processing of the mesh models was performed using MeshMixer (Autodesk, San Rafael, California, U.S.A., www.meshmixer.com). The final models were reviewed by a board-certified radiologist and by a general surgeon specialized in liver surgery (see Figs. 1, 2, 3).

### Virtual reality environment

**Fig. 4:**
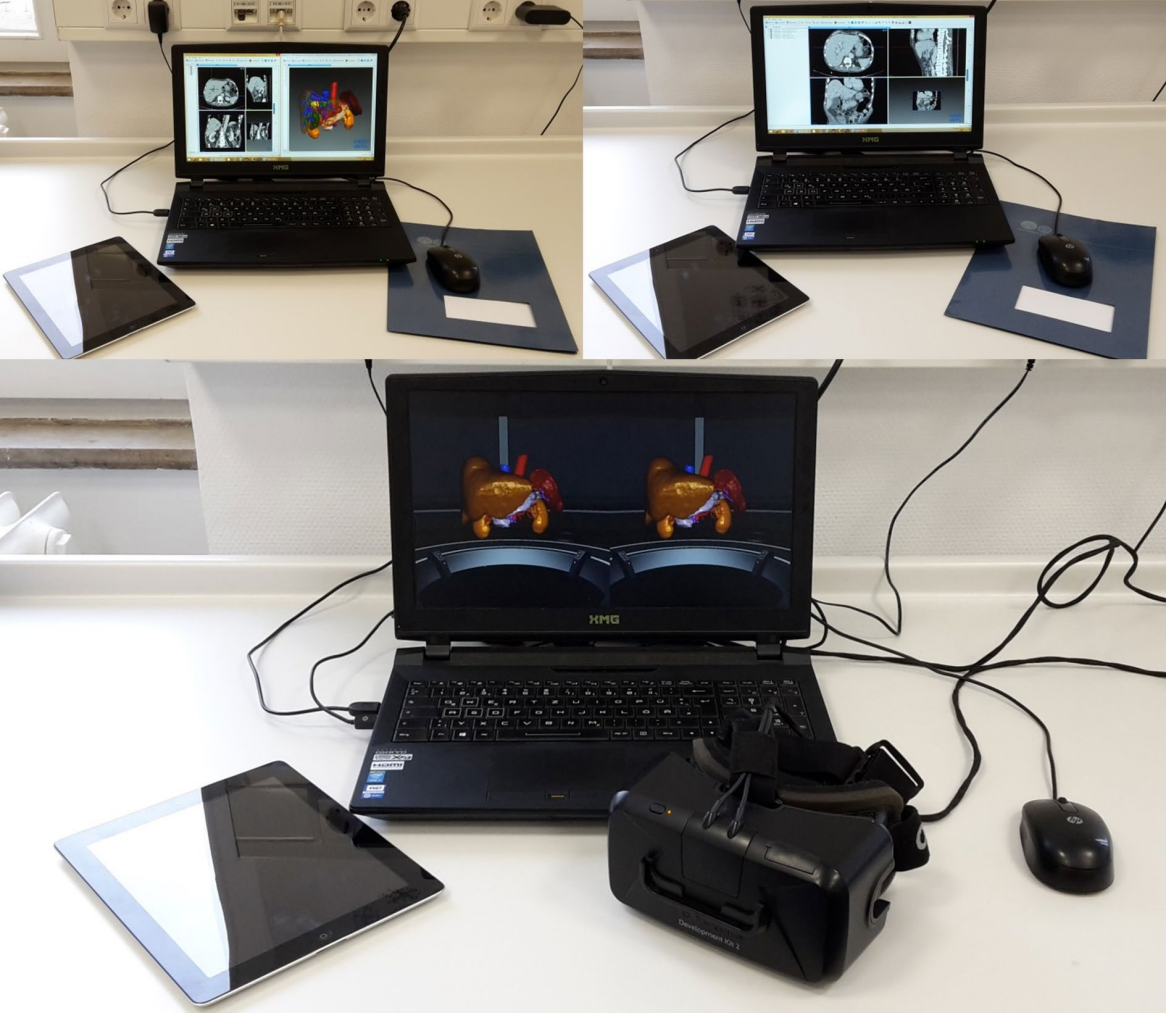
Experimental setup for both 2D (top right), 3D (top left), and VR (below) evaluation (2D shown). The laptop and VR headset were used for case evaluation. The digital tablet was used for answering the questionnaire options

For the presentation of patient information, associated anonymized computed tomography images, and 3D-models, the developed IMHOTEP-software was used (Karlsruhe Institute for Technology, Karlsruhe, Germany, www.imhotep-medical.org) (Fig. 4) [41, 45]. The software was installed on a XMG U505 computer (Schenker Technologies GmbH, Leipzig, Germany) with Intel® Core™ i7-4790S CPU with 3.20 GHz, 16 GB Rapid Access Memory and NVIDIA® GeForce™ GTX 980 M graphic card. The immersive aspect of the operation planning was realized using the virtual reality head-mounted display Oculus Rift™ (Oculus VR LLC, Menlo Park, California, USA). The Oculus Rift™ created a stereoscopic 3D perspective through its two LCD displays. When the user changed his head position and orientation, the view on the virtual scene was changed accordingly. IMHOTEP allowed the user to view the segmented 3D data, patient information and computer tomography images in this virtual reality environment.

### Interaction with the VR environment

Five Surgical views (all, tumor + vessels, tumor + arteries, tumor + veins, tumor + bile ducts) were created with predefined viewing angles, zoom and different transparency of the organs and vessels. Using these views, the users could quickly navigate to the view which was most relevant to the question at hand, or highlight vessels of interest in order to better comprehend and prepare for the individual layout of the given patient’s liver anatomy and pathology. The users could also individually use the mouse to turn and zoom the liver in free space and visualize these objects from various individual angles as well as adjust the transparency of the organs and vessels.

### Study design

This was a registered prospective, single-center, three-arm, parallel-group randomized controlled study (DRKS00011000). The study was carried out in the Department of General, Visceral, and Transplantation Surgery at Heidelberg University Hospital. Medical students at Heidelberg University Medical School during their clinical years were included. All participants received a standardized introduction covering the following topics: segmental liver anatomy, standard anatomy and anomalies of the arterial, venous and portal venous system, anatomy of the bile ducts and possible resection lines for liver surgery. Participants were randomly assigned by the investigator to one of the visualization methods (2D vs. 3D vs. VR) in a 1:1:1 ratio using the closed envelope technique with a computer-generated list for randomization. The study protocol can be viewed under the following URL: https://drks.de/search/en/trial/DRKS00011000).

All participants had unlimited time to familiarize themselves with the visualization method they were randomized to. The technical aspect of familiarization was done with a fourth patient dataset that was not used otherwise. After the introduction and familiarization, each participant then evaluated imaging data and patient information for three consecutive liver cases with increasing difficulty. In the “2D”-group, participants evaluated the imaging data in sectional views on a flatscreen monitor, patient information and labs were available on a printed sheet. In the “3D”-group, participants evaluated the imaging data as a 3D-model on a flatscreen monitor, patient information and labs were available on a printed sheet. In the “VR”-group, participants evaluated the imaging data in the VR environment, patient information and labs were integrated into this environment. Participants had to answer an 11-item-questionnaire assessing liver anatomy, tumor involvement and proposed liver resection (see Online Appendix 1). Time to answer the questions was also measured. The questionnaire was developed by board-certified surgeons with a specialization in liver surgery at the Department of General, Visceral, and Transplantation Surgery at Heidelberg University Hospital. The correct answers were defined by a board-certified radiologist and by a general surgeon specialized in liver surgery from the same institution. The correct answers for each case can be found in Table 1. After the liver cases were evaluated, participants were asked to fill out an 18-item evaluation form (see Online Appendix 2) using Likert-scales, multiple choice items and free answer options to assess the satisfaction, usefulness and potential of this system. Google™ Forms (Google Inc., Mountain View, California, USA) was used for data acquisition.

The primary outcome measure was the difference in the score (sum of correct answers) as measured by the 11-item anatomy and surgical indication evaluation questionnaire. The secondary endpoints were the time it took to answer the above-mentioned questionnaire, as well as the perceived satisfaction, usefulness and potential of the evaluated visualization method as per the 18-item evaluation form.

Continuous data was assessed using descriptive parameters (mean, standard deviation, minimum, median and maximum). Categorical data was assessed using relative and absolute frequencies. A three-group analysis using a Kruskal–Wallis-Test was carried out to compare the scores between the three groups (2D vs. 3D, 2D vs. VR, 3D vs. VR). Then pairwise comparison between the groups was performed using a Mann–Whitney *U*-Test (2D vs. 3D, 2D vs. VR, 3D vs. VR). The same comparison was used to determine statistical differences between the liver cases. A Chi-square test was be used in the case of comparing categorical data. Graphical representations of the statistical data were added whenever appropriate. The level of significance was set to 5%. Those evaluating the outcome and assessing the statistical outcome were blinded regarding to the groups. All statistical calculations were carried out using SPSS (IBM Corporation, New York, USA) software.

### Ethical approval

The study was approved by the local ethical committee in Heidelberg (S-349/2016). The trial was registered with the German Clinical Trails Register (DRKS00011000) prior to the beginning of the study. All procedures were conducted in accordance with the ethical standards of the Helsinki Declaration of 2013.

### Patient data anonymization

All patient data was retrieved in an anonymized fashion before it was integrated in the IMHOTEP-software and thus included in the study.

## Results

**Fig. 5:**
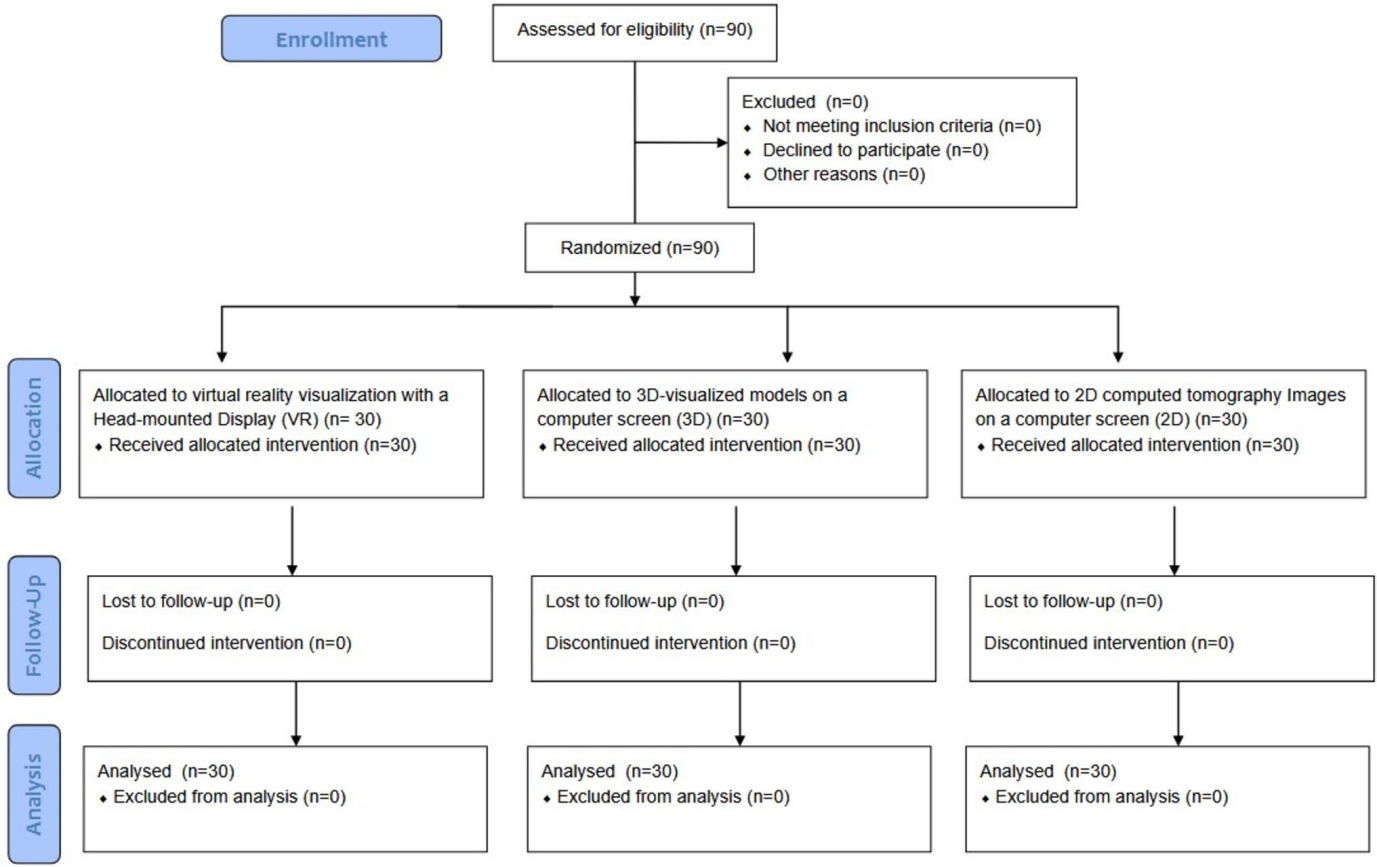
Recruitment flowchart

Between May and August 2016 90 medical students were recruited and participated in this study. Figure 5 shows the trail recruitment flowchart. Table 2 shows the statistical baseline data of the randomized study groups.

**Table 2:** Baseline characteristics of the study population

	2D group (*n* = 30)	3D-group (*n* = 30)	VR-group (*n* = 30)	*p*
Age				
Years, mean ± SD	23.9 ± 2.0	24.3 ± 2.1	23.5 ± 1.8	0.43
Sex				
Male	19 (63%)	21 (70%)	22 (73%)	0.70
Female	11 (37%)	9 (30%)	8 (27%)	
Liver operations seen				
0 operations	25 (83%)	22 (73%)	26 (87%)	0.39
> 10 operations	5 (17%)	8 (27%)	4 (13%)	
Liver operations assisted				
0 operations	30 (100%)	29 (97%)	30 (100%)	0.37
> 10 operations	0 (0%)	1 (3%)	0 (0%)	
Operating room experience				
First assistant	0 (0%)	1 (7%)	3 (10%)	0.34
Second assistant	4 (13%)	6 (20%)	6 (20%)	
Observer	26 (87%)	23 (77%)	21 (70%)	
Technologically adept (self-estimation)				
Yes/no	23 (77%)	21 (70%)	26 (87%)	0.98

### Results by visualization method

**Fig. 6:**
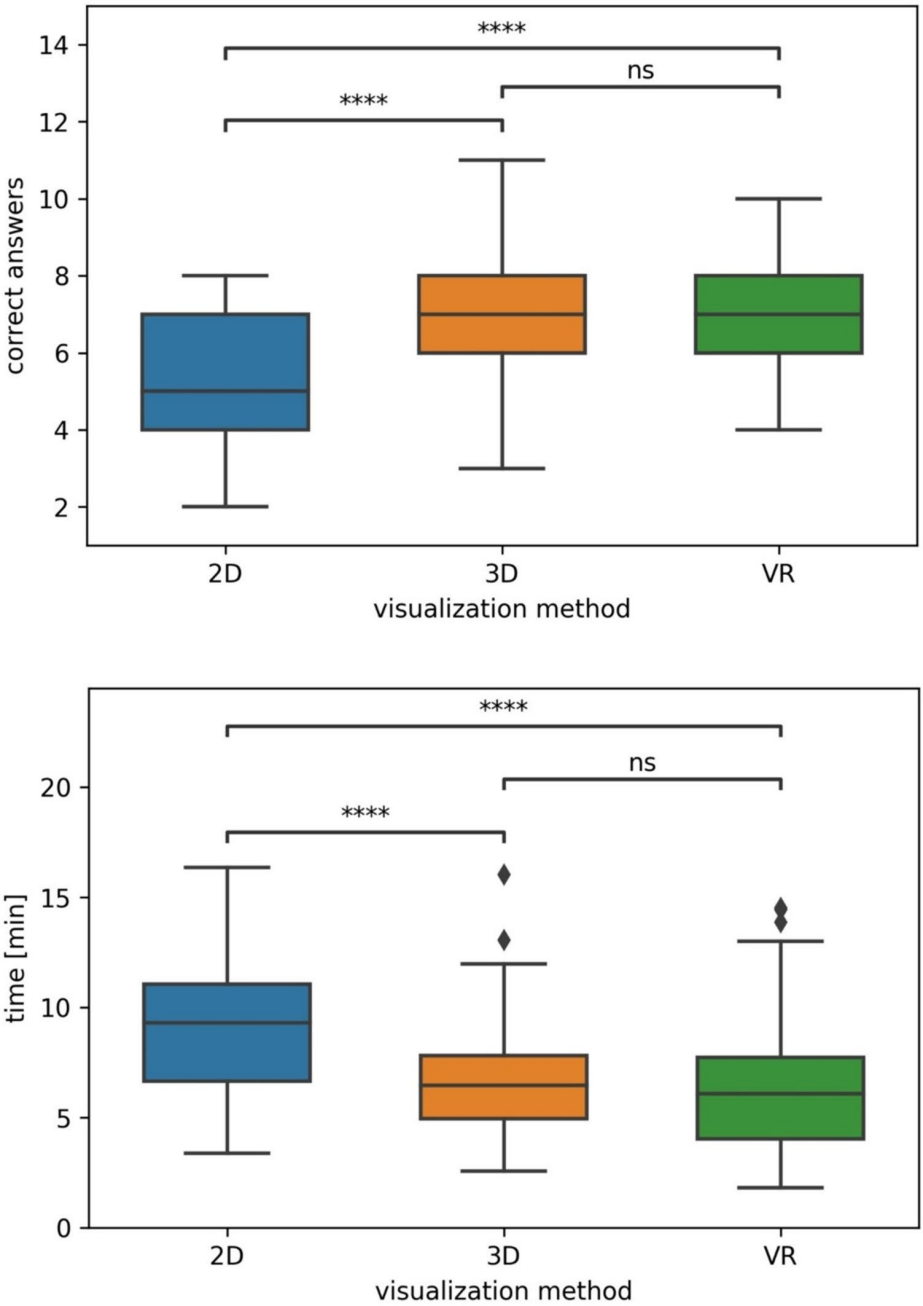
Boxplot of average correct answers and time [min] to answer by visualization method, averaged across all patient cases. *p*-value annotation legend: *ns* not significant (*p* > 0.05), *1.00e−02 < *p* ≤ 5.00e−02, **1.00e−03 < *p* ≤ 1.00e−02, ***1.00e−04 < *p* ≤ 1.00e−03, ****p ≤ 1.00e−04, Diamond symbol signifies outliers

The VR-group and 3D-group had significantly more correct answers across all cases than the 2D group (*p* < 0.001). The difference in results between 3D- and VR-groups were not statistically significant (*p* = 0.987). The VR-group and 3D-group had significantly lower time to answer across all cases than the 2D group (*p* < 0.001). The difference in time to answer between 3D- and VR-groups were not statistically significant (*p* = 0.419). Results are summarized in Fig. 6.

Analyzed by individual patient cases, the VR-group and 3D-group had significantly more correct answers than the 2D group (*p* < 0.001 for each patient). The difference in results between 3D- and VR-groups were not statistically significant in any patient case (*p* = 0.994 for patient 1, *p* = 0.827 for patient 2, *p* = 0.908 for patient 3). The VR-group and 3D-group had significantly lower time to answer per patient case than the 2D group (*p* < 0.001 for each patient). The differences in time to answer between 3D- and VR-groups were not statistically significant in any patient case (*p* = 0.823 for patient 1, *p* = 0.600 for patient 2, *p* = 0.315 for patient 3). Results are summarized in Fig. 6.

### Learning curve analysis by intragroup comparison of correct answers

In addition to comparison across groups, the patient cases were evaluated regarding differences in correct answers and answer times for each group by consecutive patient case.

In the 2D group, the sum of correct answers was significantly higher for patient 3 compared to patient 1 (*p* = 0.001) and patient 2 (*p* = 0.008). The difference between patient 1 and 2 was not statistically significant (*p* = 0.619). The decision time was significantly lower for patient 3 compared to patient 1 (*p* < 0.001) and patient 2 (*p *< 0.001). The difference between patient 1 and 2 was not statistically significant (*p* = 0.395).

In the 3D-group, the sum of correct answers was significantly higher for patient 3 compared to patient 1 (*p* < 0.001) and patient 2 (*p* < 0.001). The difference between patient 1 and 2 was not significant (*p* = 0.809). The decision time was significantly lower for patient 3 compared to patient 2 (*p* < 0.001) and patient 1 (*p* < 0.001). The decision time was significantly lower for patient 2 compared to patient 1 (*p* = 0.006).

**Fig. 7:**
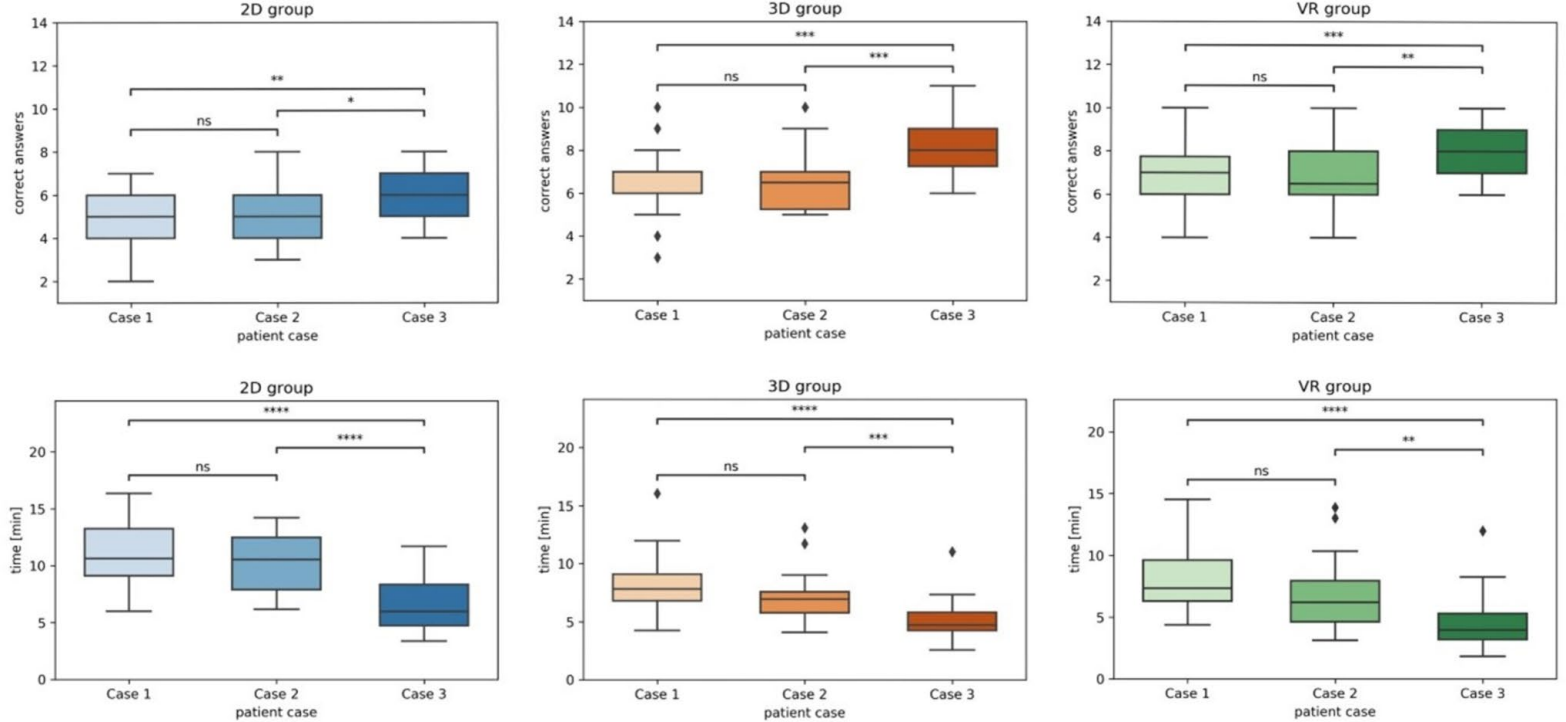
Boxplot graphs of average number of correct answers and time [min] to answer, split by study group and patient case. *p*-value annotation legend: *ns* not significant (*p* > 0.05), *1.00e−02 < *p* ≤ 5.00e−02, **1.00e−03 < *p* ≤ 1.00e−02, ***1.00e−04 < *p* ≤ 1.00e−03, ****p ≤ 1.00e−04, Diamond symbol signifies outliers

In the VR-group, the sum of correct answers was significantly higher for patient 3 compared to patient 1 (*p* < 0.001) and patient 2 (*p* = 0.001). The difference between patient 1 and 2 was not significant (*p* = 0.940). The decision time was significantly lower for patient 3 compared to patient 2 (*p* = 0.001) and patient 1 (*p* < 0.001). The decision time was significantly lower for patient 2 compared to patient 1 (*p* = 0.019). Results are summarized in Fig. 7.

### Subjective evaluation questionnaire

**Fig. 8:**
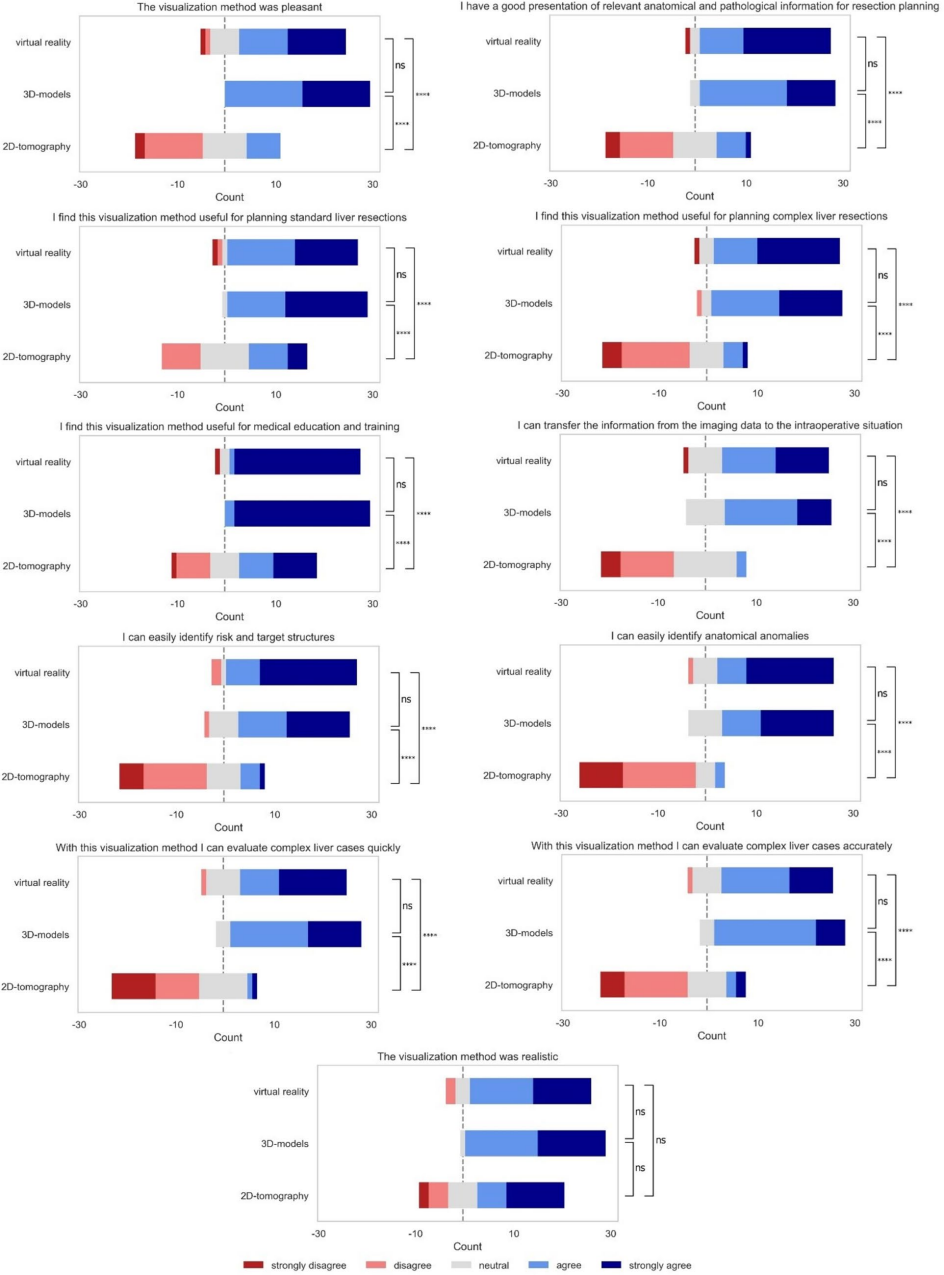
Answers to the evaluation questionnaire (Likert scale) by answer and group. *p*-value annotation legend: *ns* not significant (*p* > 0.05), *1.00e−02 < *p* ≤ 5.00e−02, **1.00e−03 < *p* ≤ 1.00e−02, ***1.00e−04 < p ≤ 1.00e−03, ****p ≤ 1.00e−04

A Mann–Whitney-U analysis of the subjective evaluation results showed that for all questions except “the visualization method was realistic”, the 2D group rated their visualization method significantly worse than both the 3D and VR-groups. For the question “the visualization method was realistic”, there were no statistically significant differences amongst all groups. In all evaluation questions, there were no statistically significant differences in the answers between the 3D and VR-group. The results for the subjective evaluation are presented in Fig. 8.

## Discussion

A VR environment with HMD can be used by surgical novices to accurately and quickly determine surgical liver anatomy and tumor involvement in complex liver cases in order to decide on an operative strategy. In the present study, the sum of correct answers in the test was significantly higher and decision time was significantly shorter with VR and 3D compared to 2D while there no differences between 3D and VR. These results were consistent in all three patient cases. In all three study groups, average performance increased per patient case, with case three having the highest number of correct answers and lowest time to answer. In the subjective evaluation, 3D and VR were preferred over 2D in all aspects. VR was evaluated as superior for the identification of anatomic anomalies, risk and target structures and for the transfer of anatomical and pathological information to the intraoperative situation compared to 3D and 2D. While 3D and VR were evaluated as being superior to 2D in overall pleasantness, for planning standard liver resections, and in medical education and training, the overall most favored visualization method was VR.

Other studies have shown 3D visualization to be advantageous in learning surgical liver anatomy with participants in 3D groups consistently answering anatomical questions more correctly and faster than 2D-groups [20, 22–25, 37]. While the cited studies mainly asked generic questions about liver anatomy with some questions regarding resections, in the present study participants were asked to answer all relevant information needed to determine an operative strategy. Jurgaitis et al. showed that 3D visualizations improved medical students’ ability to localize hepatic tumors and correctly determine the extent of the hepatic resection [25]. The present study has shown that with the help of 3D-models, surgical novices could additionally differentiate between physiological anatomy and the pathology of a patient and make a surgical decision. The 3D visualization system may facilitate the teaching of liver anatomy and pathology and could help medical students to understand the steps in deciding on the type and extent of the hepatic resection. The positive results from previous studies [20, 22–25, 37] suggest that 3D visualization and virtual reality compare favorably or may be superior to current surgical visualization teaching methods. Continued studies, e.g. focusing on other organ systems or surgical operations, may aid in establishing VR as a valid and modern surgical teaching tool [34]. Furthermore, surgical guidance systems relying on a combination of CT imaging, 3D segmentation and augmented and virtual reality systems are being rapidly developed and becoming increasingly robust [27, 39, 46–50]. This should give additional weight to the argument that medical students should receive training in, and interact with these systems, particularly in surgical fields [33, 51–53]. Surgeons may have different opinions on resectability of liver tumors depending on their expertise and experience, but also depending on their understanding of the patients’ imaging data combined with other relevant information [54]. The IMHOTEP tool may help surgical novices better understand the differences in decision-making between different surgeons. It may also help residents more quickly acquire competency in anatomical and pathological assessment of patient data [30, 55]. All three groups in the present study scored better in consecutive cases and significantly reduced the time to answer. The improvement in both speed and accuracy in all three modalities suggests a learning curve for all visualization methods. This is emphasized by the fact that even though patient 3 was deemed the most difficult patient case by the specialists, all three groups had improved their correctness score by patient 3. This adds to the argument that students should receive frequent training in such visualization systems, and demonstrates the need for continuous re-exposure to simulated or real patient cases as one of the most effective methods of learning in the medical/surgical environment.

Another argument for the marked improvement in the 3D and VR-groups may be not only because of the 3D modality, but also due to the fact that interacting with this medium is more intuitive and thus more enjoyable [56, 57]. Participants were most satisfied with VR and the VR-group was least likely to prefer a different visualization method, which again points to VR being an effective medium for keeping the subject engaged with the material, thus facilitating the learning process [40]. This satisfaction with VR training has been noted in a previous publication by Nickel et al. [58]. Pleasantness in both the 3D- and VR-group in the present study was rated very highly. However, for some users, motion sickness can be a problem arising with the use of the VR-glasses [59]. In studies examining this phenomenon, the incidence of motion sickness varies greatly and is dependent on a variety of factors, including individual susceptibility to motion sickness, duration of exposure, postural variation (standing/sitting), actions being performed, and visual motion stimulus, i.e. simulated displacement or simulated motion of the “own” virtual body [60]. The configuration least likely to cause motion sickness appears to be a short exposure time, sitting in place, and no visual motion stimuli, i.e. the virtual avatar remaining in place in concurrence with the user. As the IMHOTEP tool lacks any reason for the user to be exposed to postural variation or visual motion stimulus, the risk of motion sickness can be kept at a minimum. Variability between HMD systems has also been reported [61]. For wearers of glasses the use of VR-glasses may be awkward and make the experience less pleasant. This will likely be corrected by future improved designs of VR-glasses that accommodate for wearers of glasses.

One can argue that with better training of surgical liver anatomy and surgical decision-making, 2D radiological images might be evaluated better after a 3D/VR training period. Surgical topography can be difficult to present and visualize for novice surgeons and medical students, and the addition of 3D visualization of complex anatomy has been shown to improve learning speed and retention when compared to traditional 2D methods [20, 22, 24, 25, 62]. Surgical novices might, for example, improve their reading of CT-images if a correlation between the 3D-model and the 2D images is implemented in the software. Metzler et al. showed that training purely with 3D does not directly transfer to enhance the understanding of 2D CT-images in students [23]. However, the combination of conventional 2D images with simultaneous 3D or VR models may enable a better transfer of understanding. Further integration of conventional 2D-imaging into the IMHOTEP VR software, in order to tie it more closely to the 3D-model, is being planned and its effects will be evaluated in a future study.

The process of image segmentation is a bottleneck for 3D visualizations. In the present study, only open-source segmentation tools with semi-automatic algorithms were used for segmentation. The tools provided accurate results but the process of segmentation was time-consuming, amounting to several hours per patient case, and required board-certified surgeons and radiologists to verify the results. There are commercial segmentation services that can be used to create 3D-models from radiological images [63]. As these services continue to improve and become more commercially viable, VR integration of preoperative planning may find increasing relevance in the clinical setting.

The presented study only used standard clinical imaging modalities, freely downloadable open-source software and commercially available hardware thus enabling a cost effective and easy reproducibility. The imaging data used for this study was computed tomography images, which are generally available for surgical oncologic resection planning. The software used for creating and post-processing the segmentations and 3D-models is open-source software and can be downloaded freely (https://www.mitk.org, www.slicer.org, www.meshmixer.com). The source code for the virtual reality visualization software IMHOTEP can be downloaded freely (http://imhotep-medical.org/) and further developed as needed. A final consideration to be given to the VR environment is the aspect of telemedicine and telecommunication. As the years since the onset of the COVID-19 pandemic has demonstrated, there is an increasing demand for viable methods of digital communication at a distance, both at work and for social reasons [42, 64]. In light of the shift in many fields to remote work, and the evidence showing that this has not resulted in a loss of productivity, it appears likely that telework will remain attractive for many employers and employees even after cessation of current pandemic restrictions. Aside from the COVID-19 pandemic [44], the need for accurate telecommunication has been increasing in the medical field over the past decade [43, 65], as expert consultations e.g. in multicenter tumor boards become increasingly common, and VR solutions have been proposed for many medical applications, including remote bedside consultation, tumor board discussion and surgical planning and intraoperative guidance systems [66–69]. Especially in complex cases such as surgical liver planning, the medium should allow for accurate communication and interpretation of information, even in a remote setting. In such cases, an interactive VR platform could allow for precise discussions, for example regarding tumor location and resection possibilities, with reduced risk for error.

### Limitations

The aim of the presented study was to evaluate and explore the benefit and problems of a VR environment for training of surgical novices for liver surgery. We interpret the results that with the correct way of visualizing the clinical data even surgical novices can determined the correct liver anatomy and thus have the basis to making a decision on the right liver resection. The findings of this study do not serve to validate this tool as a surgical planning tool and cannot be generalised to surgical practitioners. The creation of the three-dimensional images by segmentation of the underlying computed tomography images is still a time-consuming process. Especially creating surgically and radiologically accurate 3D-models remains a process based on the surgical and radiological expertise and needs the validation of these experts.

## Conclusion

The findings in the present study demonstrate that three-dimensional VR visualization is a valid and viable tool for teaching surgical liver anatomy. The VR environment was preferred over the other methods by the participants and it added more enjoyment to the learning process and may thus help create a better learning effect. VR and 3D display of patient anatomy is useful for training of liver surgery for surgical novices, enabling quicker and more accurate assessment of unique patient cases and allowing for improved surgical decision-making compared to 2D display.

### Supplementary Information

Below is the link to the electronic supplementary material.

Supplementary file1 (DOCX 15 KB) IMHOTEP surgical indication evaluation questionnaire

Supplementary file2 (DOCX 15 KB) IMHOTEP evaluation questionnaire
