# Supplementary material for: Comparing a virtual reality head-mounted display to on-screen three-dimensional visualization and two-dimensional computed tomography data for training in decision making in hepatic surgery: a randomized controlled study
Source: Surg Endosc. 2024 Mar 8;38(5):2483–96. doi: 10.1007/s00464-023-10615-8 (PMC11078809; doi:10.1007/s00464-023-10615-8)
Supplement: Supplementary file 1 — Supplementary file1 (DOCX 15 KB) IMHOTEP surgical indication evaluation questionnaire [file 464_2023_10615_MOESM1_ESM.docx]

| **IMHOTEP Evaluation Questionnaire** | | |
| --- | --- | --- |
|  | Questions | Answers |
| 1. | Are you competent with computer technology? | Range from 1 (very much) to 5 (not at all) |
| 2. | How many liver resections have you seen? | 0  <10  <50  <100  >100 |
| 3. | How many liver resections have you operated on? | 0  <10  <50  <100  >100 |
| 4. | The visualization method was realistic. | Range from 1 (strongly agree) to 5 (strongly disagree) |
| 5. | The visualization method was pleasant. | Range from 1 (strongly agree) to 5 (strongly disagree) |
| 6. | Would you prefer another visualization method? | Yes, I would prefer 2D-images on a monitor  Yes, I would prefer 3D-visualization on a monitor  Yes, I would prefer 3D-visualization on Oculus Rift™  No, I am satisfied  Other: |
| 7. | With this visualization method I can evaluate complex liver cases quickly. | Range from 1 (strongly agree) to 5 (strongly disagree) |
| 8. | With this visualization method I can evaluate complex liver cases well. | Range from 1 (strongly agree) to 5 (strongly disagree) |
| 9. | Using this visualization method I have a good presentation of all relevant anatomical and pathological information for resection planning. | Range from 1 (strongly agree) to 5 (strongly disagree) |
| 10. | Using this visualization method I can easily identify anatomical anomalies. | Range from 1 (strongly agree) to 5 (strongly disagree) |
| 11. | Using this visualization method I can easily identify risk and target structures. | Range from 1 (strongly agree) to 5 (strongly disagree) |
| 12. | Overall, using this visualization method I can transfer the anatomical and pathological information from the imaging data to the intraoperative situation. | Range from 1 (strongly agree) to 5 (strongly disagree) |
| 13. | I find this visualization method useful for planning standard liver resections. | Range from 1 (strongly agree) to 5 (strongly disagree) |
| 14. | I find this visualization method useful for planning complex liver resections. | Range from 1 (strongly agree) to 5 (strongly disagree) |
| 15. | I find this visualization method useful for medical education and training | Range from 1 (strongly agree) to 5 (strongly disagree) |
| 16 | Optional: I find this visualization method useful for... | [free text] |
| 17. | Overall, I am satisfied with this visualization method. | Range from 1 (strongly agree) to 5 (strongly disagree) |
| 18. | Chose areas where improvement would be best | more Information  better Information (e.g. context sensitive)  better presentation  better interaction  better visualization  more Features (e.g. volume measurement)  no improvement necessary  Other: |
