# Supplementary material for: Comparing a virtual reality head-mounted display to on-screen three-dimensional visualization and two-dimensional computed tomography data for training in decision making in hepatic surgery: a randomized controlled study
Source: Surg Endosc. 2024 Mar 8;38(5):2483–96. doi: 10.1007/s00464-023-10615-8 (PMC11078809; doi:10.1007/s00464-023-10615-8)
Supplement: Supplementary file 2 — Supplementary file2 (DOCX 15 KB) IMHOTEP evaluation questionnaire [file 464_2023_10615_MOESM2_ESM.docx]

| **IMHOTEP Surgical Indication Evaluation Questionnaire** | | |
| --- | --- | --- |
|  | Questions | Answers |
| 1. | Please mark the segments that are affected by the tumor: | Segment 1  Segment 2  Segment 3  Segment 4a  Segment 4b  Segment 5  Segment 6  Segment 7  Segment 8  Other: |
| 2. | Is the Liver hilum affected by the tumor? | Yes  No  Other: |
| 3. | Mark the parts of the portal vein that are involved in the tumor | hepatic portal vein  left portal vein  right portal vein  right anterior pedicle  right posterior pedicle  segmental portal vein  not involved  Other: |
| 4. | Mark the hepatic veins that are involved in the tumor | right hepatic vein  middle hepatic vein  left hepatic vein  not involved  Other: |
| 5. | Is the Vena cava involved in the tumor? | Yes  No  Other: |
| 6. | Mark the parts of the hepatic artery that are involved in the tumor | right hepatic artery  left hepatic artery  middle hepatic artery  not involved  Other: |
| 7. | Mark the parts of the bile ducts that are involved in the tumor | common bile duct  common hepatic duct  right hepatic duct  left hepatic duct  hepatic duct bifurcation  Gallbladder  not involved  not visible  Other: |
| 8. | Specify which anatomical variation of the hepatic artery is present | standard anatomy  replaced right hepatic artery to the superior mesenteric artery  replaced left hepatic artery to the left gastric artery  accessory left hepatic artery from the left gastric artery  accessory right hepatic artery from the superior mesenteric artery  Other: |
| 9. | Specify which anatomical variation of the hepatic veins is present | standard anatomy  independent draining of each hepatic vein  accessory right inferior hepatic vein  Other: |
| 10. | Specify which hepatic resections are theoretically feasible | right hepatectomy  left hepatectomy  mesohepatectomy (central resection)  extended right hemihepatectomy  extended left hemihepatectomy  atypical resection  irresectable  Other: |
| 11. | Specify which hepatic resections you would favor | right hepatectomy  left hepatectomy  mesohepatectomy (central resection)  extended right hemihepatectomy  extended left hemihepatectomy  atypical resection  irresectable  Other: |
